# Tallulah Falls, North-East Georgia

**Published:** 1889-05

**Authors:** Percy N. De Duboeay

**Affiliations:** Fellow Royal College of Surgeons, Member Royal Medical Society, Member Laryngological Society of Berlin, etc., Tallulah Falls, Ga.


### TALLULAH FALLS, NORTHEAST GEORGIA.

### A Climatic Health Resort.

By PERCY N. de DUBOEAY, M. D.,

Fellow Royal College of Surgeons, Member Royal Medical Society, Member Laryngological Society of Berlin, etc., Tallulah Falls, Ga.

Tallulah Falls, Rabun county, Georgia, is now achieving such a reputation as a health resort, and its name being mentioned in so many circles, that it seems advisable to publish some data concerning the character of its climate, its effects on invalids, the accommodation offered, etc., etc., in order to meet the increasing demands for information so constantly called for by numerous enquirers.

Freeing our minds as far as we possibly can from any practical results, which we clearly observe in invalids making their residence here, it is a simple matter to show, on mere theoretical grounds, why this region and especially "Tallulah Falls," is gaining its well deserved fame as a health resort. More particularly so for consumptives. Almost all modern writers and therapeutists agree in this opinion concerning the desiderata for a climate for invalids, viz.: a certain amount of elevation above sea level, a temperature equally free from great extremes of heat and cold, a small amount of moisture of the atmosphere, without excessive degrees to cause vibrations of the throat and respiratory organs, a moderate amount of rainfall, a great number of sunny, cloudless days; besides these also qualities of a more social character, as easy access, good accommodations, a varied scenery to relieve the monotony of invalid life, and last, but not least, a friendly, sympathising people, ready to welcome and assist the weary and worried stranger who left his home, surrounded by friends and comfort, to seek health and fresh life in a distant land.

Having now pointed out the demands we ought to make from a climate which can be conscientiously recommended to the large army yearly traveling for the benefit of their health, it is an easy task to show that "Tallulah Falls" combines all these characteristics in a happy degree.

In taking up the various points separately, and turning our attention to the question of altitude first, we notice even amongst its advocates a great divergence of opinion as to how the salutary effect is obtained. We feel confident, however, in being able to show reasons towards a satisfactory explanation as to why elevated regions are preferable to low lands for invalids. There are four theories all emenated and advocated by eminent investigators in this special branch of science, who desire to prove how mountain air affects the respiratory organs. Some assert that the respiration becomes more frequent and less deep; others only speak of more frequent respiration. A third party describe them as more frequent and deeper; while the last one believes in deeper ones with their natural respiration. In our opinion none of these theories will hold good for every individual case without exception. And we do not anticipate much opposition when we give our reasons for adhering to the last explanation.

It is a mathematical thesis that in ascending heights, the mercurial column sinks in proportion to the rise in elevation. This shows that the weight of the air grows less, that the density of the atmosphere decreases; in other words, the air becomes rarefied. Therefore air in elevated regions will contain a lesser amount of oxygen in a given space, say a cubic foot, than at sea level. Generally we observe in invalids coming to a higher altitude an increase in the number of respirations. In the beginning, which is very natural, as the system tries to supply the lung with the same amount of oxygen as at sea level, and less oxygen being contained in the air, the compensation is arrived at by an increased frequency of breathing. If the elevation is not too high, and the lungs of the patient not too materially affected as to allow acclimatization, the system becomes accustomed to the changed condition of the atmosphere, and the respirations assume their normal frequency, i. e., as they were at the lowlands. But naturally and spontaneously they become deeper at the same time as the lung takes up a certain unvarying amount of oxygen to supply the blood, and has to do that in a vicarial manner by deeper inhalations, when the air is rarified. One of the reasons why there is so much controversy and disbelief amongst scientific men in regard to this point is due to the fact, that the exact amount of air consumed by an individual with one or more inhalations in mountain districts has never, to our knowledge been ascertained.

In the large physiological laboratories of our scientific centres, we find all the necessary appliances and instruments to measure the quantity of air taken up by the lungs, with a certain number of respirations in ordinary breathing. Such appliances are costly, voluminous, and not obtainable by private individuals, who do not carry on scientific pursuits on a large scale. We feel confident that if such experiments were made, the same patient would require a larger volume of air, to make the same number of respirations, or what is the same, to exhale the same amount of carbonic acid at a high altitude than at sea level. This would plainly show, that the respiratory organs have to take in a larger volume of air to satisfy its demands for oxygen, than they have to make deeper respirations to obtain the same ends, than at sea level, the very effect of these deeper respirations is a larger expansion of the chest, and this can always be achieved by healthy people, living at high elevations.

It is a known fact, that the configuration of the throat of the inhabitants of the Cordilleras, of the mountain Indians of Mexico, of the tribes of Thibet, deviate very much from what we are accustomed to see in our homes. The chests of natives inhabiting these countries grow to such an extent, are so deep and broad, that this alone signifies the larger evolutions the lung has to make, in order to supply the system with the necessary oxygen. Conceded then, that deeper respirations are required to enable the lung to perform its normal functions in mountain districts, their beneficial effects can easily be shown. Deeper inhalations expand the lung and counteract its natural tendency to contract and collapse; when we make a deep inhalation, we conduct air to parts of the lung into which the air, with a common respiration, does not fully enter. We make air pass into the finest bronchioles, and the alveoles; they gain in capacity, and if collapsed by disease, they will be re-opened and reventilated again. By a continuance of these respirations, parts already consolidated and filled with the products of chronic inflammation, will finally be reached and made accessible to the air. Under these conditions, obdurated masses, even tough secretions, the product of chronic catarrh, will be loosened and removed. The great advantage of loosening the adherance of these morbid masses, and finally freeing the lungs from them, is obvious, as this is the first step towards a successful fight of the disease. The circulation of the blood is also materially aided by the increased expansion of the lung. The carbonic acid gas escapes more readily, the blood in the affected lung can better divest itself of it, and tissue change is indirectly promoted. If we can achieve by elevated regions gradual relief of the affected lung from morbid deposits, and improve the circulation of tissue change—points which we endeavored to prove in the foregoing—we claim the right to state that altitude is one of the important features of a climate which an invalid should select for his abode.

It is with a certain amount of dread that we approach the subject of "*temperature*." All sorts of temperature having been advocated as benefitting the consumptive, and to enumerate in detail all the different opinions, and places claiming advantageous thermometrical conditions for invalids, would swell this little essay to double and triple its size. Stating briefly the well known resorts that have been recommended as examples, we will mention the dry and warm (Egypt); dry and cold ones (Minnesota); moist and warm ones (Madeira, Florida). Further equable climates, with very small diurnal or annual change of the thermometer (California); and as justly claimed, also climates with great range, as not injurious on that account (Colorado and the Western Territories); as said before, only a limited number of instances are given, but it covers climates of nearly all descriptions. Only one climate has, to our knowledge, not been recommended, and that is a cold, moist one. It is generally asserted and rightly believed, that cold moisture is dangerous to patients with pulmonary affections. We draw attention to this point, however, viz: that a population living in such a climate has for a long time been represented as a type with immunity from consumption, and later and more thorough investigation has found cold, to the present time, with only a sporadic occurrence of the disease. We refer to the Icelanders near the Arctic Circle. Of course, assuming that each advocate of a climate is writing in a *bona fide* manner, it is obvious that the question is rather a confusing one, which is only sifted by an examination of the results with patients. The endeavor now prevailing to publish reports of results at different resorts, more or less every season, is most laudable; by these it can be seen that any climate with *moderate temperature*, without excessive degrees of heat and cold, yield the longest and the best results.

To give the reader an idea of the monthly means and range of the thermometer at Tallulah Falls, dating from 1st of March, A. D. 1888 to 1st of March, A. D. 1889, we subjoin the following table:

| | MONTHS. | MEAN. | HIGHEST | LOWEST. | RAINFALL. |
|---|---|---|---|---|---|
| Year 1889. | January | 37.3 | 67 | 3 | 2.5 |
| | February | 39.1 | 69 | 6 | 4.6 |
| Year 1888. | March | 45.8 | 77 | 7 | 3.2 |
| | April | 56.2 | 83 | 33 | 2.3 |
| | May | 62.3 | 88 | 39 | 3.8 |
| | June | 70.2 | 89 | 55 | 4.1 |
| | July | 71.9 | 90 | 54 | 4.8 |
| | August | 71.3 | 89 | 52 | 4.2 |
| | September | 64.9 | 87 | 37 | 2.2 |
| | October | 54.1 | 81 | 24 | 2. |
| | November | 44.2 | 74 | 20 | 2.7 |
| | December | 39.4 | 72 | 7 | 2.5 |
| | The twelve months equals | 54.7 | 90 | 3 | 38.9 |

The above table shows greater uniformity than any other mountain section of this country; also, greater uniformity than places with the same annual mean east of the Rocky Mountains. We must refer the more scrutinizing investigator for substantiation of these data, to the reports of the chief signal officer at

Washington, D. C.; and for a detailed method of taking such observations, to a pamphlet, entitled "Asheville, the Climatic Health Resort of Western North Carolina," by the author of this little essay. Indeed the geographical position of Asheville, N. C., being so similar to that of "Tallulah Falls," the subject of this writing, that much no doubt has been rewritten, which may be found in the pamphlet on Asheville, alluded to, the same first appearing in the North Carolina Medical Journal.

The chapter on humidity of the atmosphere is more easily dispensed with, as there is a general demand for dry air amongst the medical profession as well as the public. Atmospheric moisture is usually determined by the wet and dry bulb thermometers, and from their difference the calculation is made. The usual way to speak of moisture is by naming the degree of relative humidity, which is the amount of aqueous vapor contained in the atmosphere expressed by percentage, 100 degrees being full saturation. Relative humidity therefore only gives a relative idea of the moisture of the air. The same degree of relative humidity will be felt more unpleasantly, by well and sick, when the temperature is high than when it is low, as with the change of temperature the dew point and the actual absolute amount of moisture changes. Air at a higher temperature holds more water suspended in a given volume of air than air at a lower temperature, both having the same degree of relative humidity. The relative atmospheric humidity of Tallulah Falls for the year ending 1st March, 1889, was 69.3° per cent. If we take 54 degrees temperature as Tallulah's annual mean, and 60 per cent its relative humidity, we find about 3.9 grains of aqueous vapor contained in a cubic foot of air, whilst with 69 per cent relative humidity, and 80 degrees temperature, we have 9.9 grains of vapor in a cubic foot.

The amount of vapor the lungs exhale with each respiration has been shown by physiological experiment to be nearly the same whatever air we breathe. It is obvious that more moisture will be drawn from the lung, when we inhale an air 3.9 of vapor, than one containing 9.9 grains in a cubic foot. If dryness of the air is therefore claimed for a place, we ought not to be satisfied with data of relative humidity alone, but should extend our investigation to the absolute humidity before we form a definite opinion. The almost general neglect of not considering the absolute humidity is in our opinion the only reason why the climate of Florida could be called a "not moist one!" its average relative humidity being about 70 per cent, with a few degrees ranging more or less. This figure is not high especially when compared with many European resorts, but its mean temperature being high, the *absolute* amount of vapor in the air is large. Where the gray ball moss, "Tillandria," grows to any extent, a large amount of moisture is beyond doubt contained in the atmosphere. The relative humidity, as well as the rainfall at Tallulah Falls are less in the colder months than in the warmer. For instance the humidity at Tallulah for the third quarter of the year 1888, A. D., was 81.4 per cent, whilst for the fourth quarter of the same year it was only 64.3 per cent. The rainfall for the third quarter of the year 1888 was 11.33 inches, whilst the cold quarter of the year, viz: the fourth, gave 4.94 inches of rainfall. From these figures the greater dryness of the winter months is apparent, and a natural consequence of this state of the atmosphere is a great number of clear, cloudless days in winter. The effect of bright, genial sunshine on invalids cannot be too highly valued. The natural beautiful scenery of this section of country appears then in its full glory, and the eye never gets tired looking at the variety of mountains, towering in one grand continuous range from south to north. Our pen is not skilled enough to do justice to this beauteous and romantic country. Many able writers have pictured and praised its wonders. Here exist the famed water Falls of Tallulah, often termed the Niagara of the South. Such a variety of landscape scenery goes far to counteract the monotony of invalid life, which otherwise grows more and more on patients the longer they remain in any one place. This pleasing little town, for so many years known only as a summer pleasure resort, now blossoming by the will of God, through his infinite mercy, into a resort for the invalid world, is unequalled outside of the great cities for its hotels and general accommodations. Three large hotels are in existence, supplied with every comfort, and blessed with running spring water, brought direct from the mountain sides through piping.

During the hottest midsummer weeks the nights are always cool, and a blanket on the bed is ever acceptable

Picturesque cottages are being built by visitors from North and South, who spend much of the year here. Indeed the day is not far distant when this "Alpine gem," Tallulah Falls, will have become as fashionable a resort for the indolent wealthy classes, as famed Saratoga or Long Branch in the North is to-day. And no place on this gigantic continent can ever surpass it in the life-giving elements, especially to the consumptive.

Here, when a few more years have rolled away, and the true value of that word "altitude" is more generally understood, will come thousands of sufferers annually. The seed is already sown, and the ground is rich. It only remains now for the husbandmen to come and reap the crop of health, and with health, happiness, which Tallulah offers so plentifully and so truly to all people.

Visitors to Tallulah Falls, traveling either from the North or South, pass over the Air Line railroad, changing cars at "Cornelia" (formerly known as Rabun Gap Junction). The distance from Cornelia is a twenty-one mile run to Tallulah, over a road-bed unequalled for its smoothness, and perfection of detail in every particular. Indeed, Judge Bailey Thomas, the owner and manager of this, the Blue Ridge & Atlantic Road, has left no stone unturned to assure both comfort and absolute safety to the voyager who, whether traveling for his health's sake, or on pleasure bent, finds his ideal realized at Tallulah Falls.

